# Seizure occurring with retinal laser therapy: a report of the first case with frequency-doubled Nd-YAG

**DOI:** 10.5935/0004-2749.20220062

**Published:** 2025-02-11

**Authors:** Cassiano Mateus Forcelini, Roger William Cruz de Syllos, Mônica Manica, Gabriel Mello Mattos Terra, Alana Cardozo Macagnan, Bárbara Colombo, Eduardo Muniz Dudzig

**Affiliations:** 1 Faculdade de Medicina, Universidade de Passo Fundo, Passo Fundo, RS, Brazil

**Keywords:** Retinal diseases, Retinal detachment, Laser therapy/ adverse effects, Seizure, Epilepsy, Epilepsy; reflex, Doenças retinianas, Descolamento retiniano, Terapia a laser/efeitos adversos, Convulsão, Epilepsia, Epilepsia reflexa

## Abstract

Laser photocoagulation is a safe method for the treatment of retinal disorders. We present a case of a 21-year-old woman with high myopia, retinal detachment in the right eye, and bilateral lattice degeneration. She underwent surgical repair in the right eye followed by bilateral retinal laser therapy. During laser photocoagulation of the left eye, she experienced a generalized tonic-clonic seizure for the first time in her life. She had a positive family history of epilepsy. Neurological examination and brain magnetic resonance imaging findings were normal, but an electroencephalogram revealed epileptogenic discharges, more frequent during photostimulation. She avoided flickering lights during the 2-year follow-up, without seizure recurrence. Approximately 5% of patients with epilepsy have photosensitive epilepsy, of whom a considerable proportion will experience seizures only during exposition to flashing lights. Laser photocoagulation was already successfully employed in an animal model of photosensitive epilepsy. Personal or family history of photosensitivity warrants a neurological consultation before retinal treatment with laser therapy.

## Abstract (pt)

Fotocoagulação a laser é método seguro para tratamento de retinopatias. Apresentamos o caso de uma mulher de 21 anos com alta miopia e degeneração lattice bilateral que sofreu descolamento de retina no olho direito e foi submetida a tratamento cirúrgico e ulterior laserterapia. Durante a fotocoagulação no olho esquerdo, ela teve uma convulsão tônico-clônica generalizada, a primeira em sua vida. Havia história familiar de epilepsia. O exame neurológico e a ressonância magnética de en céfalo foram normais, mas o electroencefalograma revelou descargas epileptogênicas, mais frequentes durante a fotoesti mulação. Ela evitou luzes piscantes durante os 2 anos subsequentes, sem recorrência de convulsões. Cerca de 5% dos pacientes com epilepsia têm fotossensibilidade. Proporção considerável deles terá convulsões somente durante exposição à luz piscante. Fotocoagulação a laser já foi empregada como modelo animal de sucesso para epilepsia fotossensível. Presença de fotossensibilidade na história pessoal ou familiar deve merecer avaliação neurológica antes do tratamento retiniano.

## INTRODUCTION

Laser photocoagulation is a method of treatment for retinal disorders, with complications virtually limited to the eyes. Choroidal detachment, exudative retinal detachment, narrowing of the anterior chamber angle, transient glaucoma, and macular edema were frequently reported with gas-based lasers, most commonly with argon^(1)^. In the early 2000s, gas-based lasers were replaced by other techniques employing diode, diode-pumped solid state, and optically pumped semiconductor lasers, which are nowadays described as “conventional”^(2,3)^. With the advent of frequency-doubled neodymium-do ped yttrium aluminum garnet (Nd-YAG) laser, the pulse duration became shorter compared with previous techniques, leading to less transmission of thermal energy to the choroid and allowing for a simultaneous application of multiple laser spots^(1)^. Consequently, laser burns are lighter and smaller than those with gas-based and conventional methods, resulting in a faster and more comfortable procedure for patients^(2,3)^.

In the 1980s, 2 cases of generalized seizures induced by argon-based laser therapy were described, representing the only reports of systemic complications caused by retinal laser burns^(4,5)^. We report the third case of generalized tonic-clonic seizure during retinal laser photocoagulation treatment. To the best of our knowledge, this is the first report of this complication secondary to the use of frequency-doubled Nd-YAG laser. We discuss the recommendations for predicting seizure in patients with previous photosensitive epilepsy and a family history of epilepsy, since most patients presenting with photosensitivity seizures have no prior personal history.

## CASE REPORT

A 21-year-old Caucasian woman with high myopia sought medical assistance due to a visual spot in the right eye in the last 7 days. Fundoscopic examination revealed inferior retinal detachment in the right eye, associated with bilateral lattice degeneration. Retinal detachment was confirmed by ocular ultrasound.

The patient underwent a surgical repair of the retinal detachment (scleral buckling). After 1 month, she was subjected to pars plana vitrectomy with silicon oil due to a new inferior retinal detachment. Two months later, she underwent peripheral circumferential retinal scatter photocoagulation of the left eye, following a peribulbar block with 9 mL of ropivacaine (1%) with 1 mL of hyaluronidase (300 IU ml^-1^). Direct frequency-doubled Nd-YAG lasers (wavelength, 532 nm) (Vitra, Quantel Medical, Cournon d’Auvergne, France) were used in a pulsed mode with shots of 0.3 seconds, intervals between the shots of 0.2 seconds, and a power of 250 mW. After 3 minutes of laser therapy, the patient suddenly developed a generalized tonic-clonic seizure lasting 2 minutes, followed by a 15-minute postictal state. Laser photocoagulation was discontinued as soon as the seizure started. The patient reported no complaints after the event but remained under close observation for the subsequent hour, exhibiting normal vital signs and normal blood glucose levels. She denied previous seizures and was otherwise healthy. She regularly took only contraceptive pills. Her parents reported a family history of epilepsy in a second-degree relative in childhood. A neurologic consultation was recommended, but the patient insisted on resuming the laser therapy to complete the ophthalmological treatment prior to consulting a neurologist. An additional session of laser photocoagulation lasting 10 minutes was undertaken on the same day without complications.

A few days later, the patient was assessed by a neurologist. She had no recurrence of seizure. The neurological workup, including brain magnetic resonance imaging, revealed no abnormalities. An electroencephalogram during wakefulness revealed normal basal rhythm with several short bursts of generalized sharp waves, more frequent during flashing light stimulation (5 Hz).

The diagnosis of genetic generalized and pure photosensitive epilepsy was established. The patient was recommended to avoid flashing lights and to take valproic acid (250 mg twice daily). She stopped medical therapy 6 months later, while adhering to the recommendation on flickering lights. At 2 years, she reported no recurrence. Electroencephalography is scheduled for the next visit.

## DISCUSSION

A seizure during retinal laser photocoagulation is an uncommon event in ophthalmological practice. Although rare, it is unlikely to be a coincidence. Moreover, it can even be anticipated in patients with a personal or family history of photosensitive epilepsy.

Approximately 5% of patients with epilepsy have photosensitive epilepsy, which is more common in younger individuals and in women^(6)^. Photosensitive epilepsy is the most common type of reflex epilepsy in humans. Photosensitivity, which is the hallmark of photosensitive epilepsy, is described as an abnormal electroencephalogram response to visual stimuli known as the photoparoxysmal response^(7)^.

A considerable proportion of patients with photosensitive epilepsy will experience seizures only during exposition to several forms of flashing light, including retinal laser photocoagulation. By the way, such event does not come as a surprise, because photocoagulation of the macula and peripheral retina has been successfully employed in an animal model of photosensitive epilepsy in primates^(8)^.

Patients considered for laser photocoagulation should be asked about personal history of seizures and antiepileptic treatment before therapy. Neurological consultation or even antiepileptic treatment before retinal photocoagulation should be considered if there is any indication of photosensitivity in medical history. The main type of photosensitive epilepsy is genetic (idiopathic) generalized epilepsy, a condition that develops in young age, has a high rate of family recurrence, and is more common in women^(7)^. Thus, another patient profile that warrants attention because of the potential risk of seizures during laser treatment includes young age, female sex, and a family history of early-onset epilepsy. This may have been the case of our patient, who fulfills one of the current diagnostic criteria for epilepsy: a single episode of seizure but with a high risk of recurrence^(9)^ based on considerable abnormalities on electroencephalogram. Photosensitive seizures are usually benign and cease spontaneously, without the need for emergency antiepileptic treatment. The main concern is to avoid trauma during seizure (e.g., a fall).

It is unlikely that the seizure in our patient was triggered by the anesthetic procedure. Ropivacaine has reduced the risk of cardiovascular and neurological toxicity compared with other local anesthetics that are commonly used for peribulbar anesthesia^(10)^.

Retinal laser photocoagulation can induce seizures in patients with photosensitive epilepsy, although the probability is low. This may be predicted to some extent by a careful assessment of the personal and family history of seizures, especially those induced by flickering lights.
